# Enhancing potassium availability and dynamics in some Egyptian soils through biochar application

**DOI:** 10.1038/s41598-026-36281-z

**Published:** 2026-02-13

**Authors:** Muhammad Ayman

**Affiliations:** Department of Water and Soil Sciences, Faculty of Technology and Development, Zagazig University, Zagazig, 44519 Egypt

**Keywords:** Biochar, Potassium dynamics, Thermodynamic parameters, Gibbs free energy, Potential buffering of K, Egyptian soils., Ecology, Ecology, Environmental sciences, Plant sciences

## Abstract

Potassium (K) deficiency is a major constraint to crop productivity in Egyptian soils, particularly in coarse-textured soils. In pot experiments, the study evaluated impact of application of biochars at a rate of 3% (w/w) produced at 450 °C for 4 h, from four agricultural residues (sugarcane bagasse residues biochar (SBR), olive stone pomace biochar (OSP), orange fruit pomace biochar (OFP), and maize stover residues biochar (MSR)) on K availability, K dynamics, and specific soil physicochemical properties across four Egyptian soil types (sandy, loamy, clayey, and calcareous), in addition to its effects on wheat growth. Biochars varied in surface area (23.72–41.82 m² g⁻¹) and nutrient content, with MSR showing the highest plant available nutrients, while OSP exhibited the highest cation exchange capacity (56.78 cmol _(+)_ kg⁻¹). Application of biochars increased soil water-holding capacity (WHC) by 17–35.5%, cation exchange capacity (CEC) by 18–163%, depending on soil type. Thermodynamic parameters of K were significantly improved; labile-K (K_L_) increased by 103.6% in sandy soil with MSR, 59.01% in loamy soil with OSP, 48.55% in clayey soil with SBR, 849% in calcareous soil with MSR. Activity ratio of K at equilibrium ($\:{\mathrm{AR}}_{0}^{\mathrm{K}}$) increased by 33.33% in sandy soil with MSR, 20.00% in clayey soil with SBR, 75.0% in calcareous soil with MSR, while in loamy soil decreased by − 16.66% in loamy soil with OSP. Potential buffering capacity of K (PBC^K^) increased with application of MSR by 96.99%, 119.89%, 45.90% in sandy, loamy and clayey soils, while PBC^K^ increased by 421.39% with OSP application in calcareous soil. Gibbs free energy (ΔG) became more negative (up to − 5.565 kcal mol⁻¹), and Gabon selectivity coefficient (K_G_) increased by 82.5%. Wheat fresh and dry biomass increased by 25.9–84.6% and 16.9–63.8%, respectively. Uptake of N, P, and K on wheat tissues increased by 27.3–142.2%, depending on biochar type and soil. In general, biochar-amended treatments produced higher wheat biomass and nutrient uptake than the unamended controls, with MSR demonstrating the most consistent performance across different soil types, followed by OSP. These findings highlight the importance of matching biochar type with soil characteristics to optimize K availability, improve K-use efficiency, reduce reliance on mineral fertilizers, and support sustainable soil fertility management under arid and semi-arid conditions.

## Introduction

Potassium (K) is an essential macronutrient for plant growth, crop productivity, and resistance to environmental stress^1–5^. Potassium occurs in several pools in soil phases, including mineral, organic, non-exchangeable, and exchangeable forms, which remain in a dynamic continuous equilibrium^6,7^. Understanding K dynamics is therefore important for estimating the soil K supplying and replenishment capacity over the long term. Such estimations are commonly based on thermodynamic indicators that describe K behavior in soils^2,3,8^. The quantity–intensity (Q–I or ∆K${\mathrm{AR}}_{{}}^{{\mathrm{K}}}$) relationship is widely used to evaluate soil K availability and buffering capacity^1,2^. Various parameters calculated from this Q-I relationship, such as Labile-K (K_L_), activity ratio of K at equilibrium ($\:{\mathrm{AR}}_{0}^{\mathrm{K}}$), potential buffering capacity of K (PBC^K^), Gibbs free energy (∆G), are useful for describing K retention, release, and equilibrium status under different soil management practices^1,2,9^.

Increasing food demand has intensified agricultural activities, leading to continuous removal of nutrients from soils. As a result, K deficiency has become a limiting factor for crop production in many regions^10–12^. Low K availability has been reported particularly in Egyptian sandy, loamy, and calcareous soils^1,3,13^. Although K deficiency can be corrected by applying mineral K fertilizers, this practice increases production costs. Moreover, excessive fertilizer application may negatively affect soil quality and the surrounding environment^12,14,15^. These limitations encourage the search for alternative and more sustainable approaches to improve K availability in soils.

Large amounts of agricultural and agro-industrial residues are generated from fruit juice processing, olive oil extraction, sugarcane production, and cereal cropping systems. When not properly managed, these residues accumulate in the environment or are disposed of by open burning, contributing to pollution and greenhouse gas emissions^1,16^. Converting agricultural residues into biochar provides a practical and environmentally safe management option. Biochar is a stable, carbon-rich material produced through the pyrolysis of biomass under limited oxygen conditions^1^. The properties and agronomic effectiveness of biochar depend strongly on feedstock type and pyrolysis temperature^1,16^. Several studies have shown that biochar application can improve soil properties and nutrient retention, particularly in nutrient-poor soils^5,16–18^.

In addition to its effects on soil physical and chemical properties, biochar has been reported to enhance nutrient availability, especially K, through increases in cation exchange capacity and water-holding capacity^16,19–21^. Previous studies also demonstrated that biochar amendments can improve K uptake by plants, indicating their potential as a partial substitute for mineral K fertilizers^1,5,16,19–21^. However, despite the growing number of biochar studies, information remains limited regarding the effects of biochars produced from different agricultural residues on K dynamics, thermodynamic parameters, and buffering capacity across different soil types. This gap is particularly evident for Egyptian soils, where studies addressing K equilibrium and Q–I-based thermodynamic parameters following biochar application are still scarce.

Therefore, this study hypothesized that biochar type and soil texture interactively impact K availability, thermodynamic parameters and nutrients uptake by wheat plant. The main objective of this work was to evaluate K availability, K thermodynamic parameters, and selected physicochemical properties of four investigated Egyptian soils (sandy, loamy, clayey, and calcareous soils), as well as wheat growth grown in these studied soils after application of different biochars (sugarcane bagasse residues biochar SBR, olive stone pomace biochar OSP, orange fruit pomace biochar OFP, and maize stover residues biochar MSR) at a rate of 3% (w/w). This study will help several groups, including researchers, farmers, agronomists, and policymakers seeking sustainable strategies to safe use of waste, enhance soil fertility, optimize K use, and improve plant growth under arid and semi-arid conditions.

## Methods

### Soil samples and locations

Soil samples were obtained from several locations owned by some local farmers in Egypt from a top layer (depth = 0–30 cm). These soils include sandy soil (Ismailia Governorate), loamy soil (Belbis, Sharkia Governorate), clayey soil (Zagazig, Sharkia Governorate) and calcareous soil (El-Nubaria, Beheira Governorate). The permission was obtained from some Egyptian farmers verbally to collect these soil samples. Samples were air-dried, and sieved with a 2 mm mesh size. The initial characteristics and descriptions of the studied soils present in Table 1.

**Table 1:** Initial characteristics of the investigated soils.

Property	Sandy soil	Loamy soil	Clayey soil	Calcareous soil
Particles size distribution (g hg^− 1^)	–	–	–	–
Sand	80	40	32	25
Silt	15	38	18	60
Clay	5	22	50	15
Texture*	Sandy	Loamy	Clayey	Silty loam
Soil order**	Inceptisol	Entisol	Vertisol	Aridisol
pH	7.84	7.62	7.51	8.19
EC (dS m^–1^)	1.69	2.12	2.67	2.85
CaCO_3_ (g kg^–1^)	9.64	28.45	21.29	197.57
Organic matter (g kg^–1^)	8.21	11.69	12.34	9.31
CEC (cmol kg^–1^)	11.74	18.64	28.19	12.64
KCl-N (mg kg^− 1^)	15.54	18.69	21.85	18.54
NaHCO_3_-P (mg kg^− 1^)	7.11	12.57	13.12	8.18
1 M NH_4_OAc–K (cmol kg^− 1^)	0.097	0.372	0.41	0.309

*Soil texture was classified according to the texture triangle of the USDA, and **these soils were classified according to soil survey staff^51^.

### Preparation of applied biochars

Four biochars were produced sugarcane bagasse residues biochar (SBR), olive stone pomace biochar (OSP) after oil extraction, orange fruit pomace biochar (OFP) after juice extraction, and maize stover residues biochar (MSR). Each of the materials was air-dried, then, they were all subjected to pyrolysis within a muffle furnace at a temperature of 450 °C for 4 h with limited oxygen^22–25^. After the biochars had been cooled to room temperature, they were all mechanically ground to produce uniformly sized particle biochars less than 0.2 mm. Standardization of the biochar samples enabled easy comparative assessment of the impact of the various biochars on soil properties, potassium, as well as wheat growth.

### Experimental design, Biochar application and wheat cultivation

Four separated pot experiments followed a completely randomized design (CRD), with one factor (biochar type). Four biochars were applied at a uniform rate of 3% (w/w) for each soil. For each soil, five treatments were applied to the four types biochar and replicated three times as follows;

- sandy soil (S): Control, SBR, OSP, OFP and MSR treatments,
- loamy soil (L): Control, SBR, OSP, OFP and MSR treatments,
- clayey soil (C): Control, SBR, OSP, OFP and MSR treatments,
- calcareous soil (Ca): Control, SBR, OSP, OFP and MSR treatments (for each soil: five treatment × three replicates = 15 plots, in total 60 plots).

For each soil type, fifteen plastic pots were filled with 3 kg of the prepared soil only (control treatment) and soil with biochar (3% biochar, 90 g biochar /pot). Wheat variety (*Triticum aestivum* L., local variety Giza 91) was obtained from the Agricultural Research Center in Giza City (NRC), Egypt. Wheat grains were sown on the 6th of November, 2023. Fifteen days after germination, seedlings were thinned to five plants per pot. After 50 days of sowing, wheat plants were harvested, washed with distilled water, and air-dried for two days. Wheat samples were dried at 70 °C for 72 h, ground to a fine powder using a stainless-steel mill and coded for chemical analysis^26^.

### Laboratory analyses of soil, Biochar and plant samples

Soil texture was determined using the hydrometer method. Soil pH was measured in a saturated paste using a pH meter, while electrical conductivity (EC) was determined in the soil paste extracts. Organic matter (OM) was determined via the dichromate oxidation method (H₂CrO₇), while calcium carbonate (CaCO₃) content was obtained by the back titration method^26^. Available nitrogen (N) was extracted using KCl and determined by the Kjeldahl method. Soluble potassium (K^+^), calcium (Ca²⁺) and magnesium (Mg²⁺) ions of extracts were measured by flamephotometer and titration with EDTA-Na₂, respectively, according to Estefan et al. (2013). Cation exchange capacity (CEC) of studied soils was determined using 1 M NaOAc method, while available-P was extracted with the 0.5 M NaHCO₃ and determined colorimetrically. Exchangeable-K was measured using flamephotometer. Extractable-K from both soils and biochars was assessed using distilled water and 1 M NH₄OAc^26^.

Biochar acidity (pH) and electrical conductivity (EC) were measured in 1:5 (w/v) biochar-to-water suspensions according to Pandian et al. (2016). Soluble ions, including potassium (K^+^), calcium (Ca²⁺) and magnesium (Mg²⁺) of extracts were measured by flamephotometer and titration with EDTA-Na₂, respectively, according to Estefan et al. (2013). Cation exchange capacity (CEC), and water-holding capacity (WHC) were determined by NaOAc extraction, and the funnel method, respectively, as described by Estefan et al. (2013). Available some macronutrients (N, P, K) were extracted using KCl, NaHCO₃, and NH₄OAc solutions, respectively^26^. Total N, P, and K of biochars and plant samples were determined after wet digestion with H₂SO₄–H₂O₂, followed by Kjeldahl, colorimetric, and flame photometric methods, respectively^26,27^. The specific surface area of biochars was measured using the BET method (Autosorb AS-1MP, Quantachrome, USA). Fourier transform infrared (FTIR) spectroscopy was performed to identify functional groups in selected biochar samples using a PerkinElmer FTIR spectrophotometer.

### Calculations of thermodynamic parameters of K

The exchange equilibrium of K between the soil solid phase and liquid phase was investigated following the method described by^28^. Five grams of oven-dried sandy soil were shaken with 50 mL of 0.01 M CaCl₂ solution (used as a background electrolyte) containing increasing KCl concentrations of 0, 0.1, 0.2, 0.5, 1, 1.5, 3, and 5 mM. The suspensions were agitated for three hours, left to equilibrate for 24 h, and then filtered for analysis. Activity of K and thermodynamic parameters of K were calculated according to the following equations:

1. Ionic strength (*I*) was calculated according to Griffin and Jurinak^29^:
  1 $${\mathrm{I}}=0.0129{\mathrm{EC}},$$
  where: *I* is ionic strength, *EC* is electrical conductivity (dS m^− 1^) .
2. Activity coefficient of K (*Log γi*) was calculated according to the extended Davis’s equation as:
  2 $$\log \gamma i= - AZ{i^2}\frac{{\sqrt I }}{{\left( {1+\sqrt I } \right)}} - 0.3I,$$
  where Log γ_i_ is the activity coefficient of K, *A* = 0.5092, *Z*_*i*_ is a charge of the ion, and I is ionic strength (mol L^− 1^).
3. Activity of K^+^ (***a***_*i*_):
  3 $${a_i}\left( {{\text{mol }}{{\mathrm{L}}^{ - {\mathrm{1}}}}} \right)={{\mathrm{g}}_{\mathrm{i}}}{{\mathrm{m}}_{\mathrm{i}}},$$
  where: *a*_*i*_ is K − activity, γ_i_ is coefficient of K − activity, and m_i_ is a concentration of K (M).
4. Activity ratio of K^+^ (${\mathrm{AR}}_{{}}^{{\mathrm{K}}}$):
  4 $${\mathrm{A}}{{\mathrm{R}}^{\mathrm{K}}}({(mol{\text{ }}{L^{ - 1}})^{0.5}})=\frac{{aK}}{{\sqrt {(aCa} +aMg)~}},$$
  Where: *aK* is K − activity, *aCa* is Ca − activity, and aMg is Mg − activity.
5. Potential buffering capacity of K was calculated according to Sparks^30^,
  5 $${\mathrm{PB}}{{\mathrm{C}}^{\mathrm{K}}}({\text{cmol k}}{{\mathrm{g}}^{ - {\mathrm{1}}}}/{({\text{mol }}{{\mathrm{L}}^{ - {\mathrm{1}}}})^{0.{\mathrm{5}}}})=\frac{{\Delta {\mathrm{K}}}}{{\Delta {\mathrm{A}}{{\mathrm{R}}^{\mathrm{K}}}~}},$$
6. Gibbs Free energy (∆G) was calculated according to Woodruff^31^
  6 $$\Delta {\text{G }}\left( {{\text{kcal mo}}{{\mathrm{l}}^{ - {\mathrm{1}}}}} \right)=--{\text{RT lnAR}}_{0}^{{\mathrm{K}}},$$
7. Gabon selectivity coefficient (K_G_ or relative affinity).
  The Gapon selectivity coefficient (K_G_) was calculated as a simplified form of the classical Gabon relationship to provide a comparative indicator of K retention strength among treatments, following the conceptual linkage between PBC^K^ and CEC described by Sparks^32^, Zörb et al.^33^, and Wang et al.^7^.
  7 $${{\mathrm{K}}_{\mathrm{G}}}=\frac{{{\mathrm{PBC}}~\;{\mathrm{of}}\;{\mathrm{K}}}}{{{\mathrm{CEC~}}}}.$$

### Statistical analysis

Data for each soil type were analyzed separately using one-way analysis of variance (ANOVA) based on a completely randomized design (CRD). The assumptions of ANOVA were checked prior to analysis. Mean differences among treatments were separated using the least significant difference (LSD) test at *p* ≤ 0.05. All statistical analyses were performed using R software (version 4.3.3), and figures were prepared using Microsoft Excel 365 and Origin (version 9).

## Results

### Evaluation of the applied biochars

**Fig. 1:**
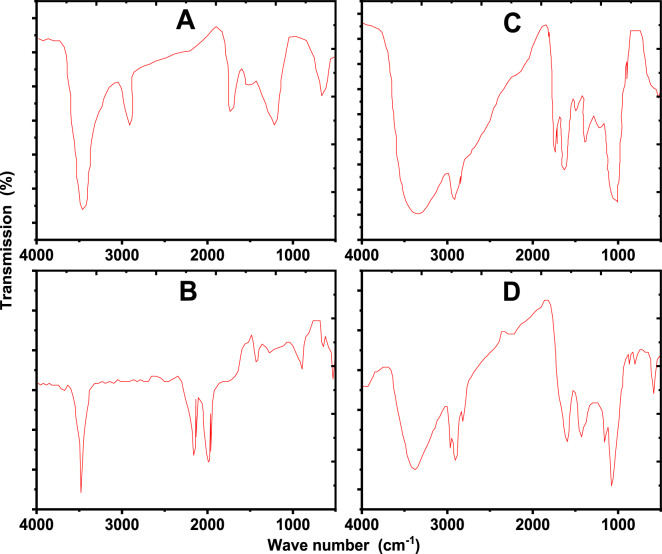
FTIR of applied biochars (sugarcane bagasse residues (**A**), olive stone pomace (**B**), orange fruit pomace (**C**) and maize stover residues (**D**).

**Fig. 2:**
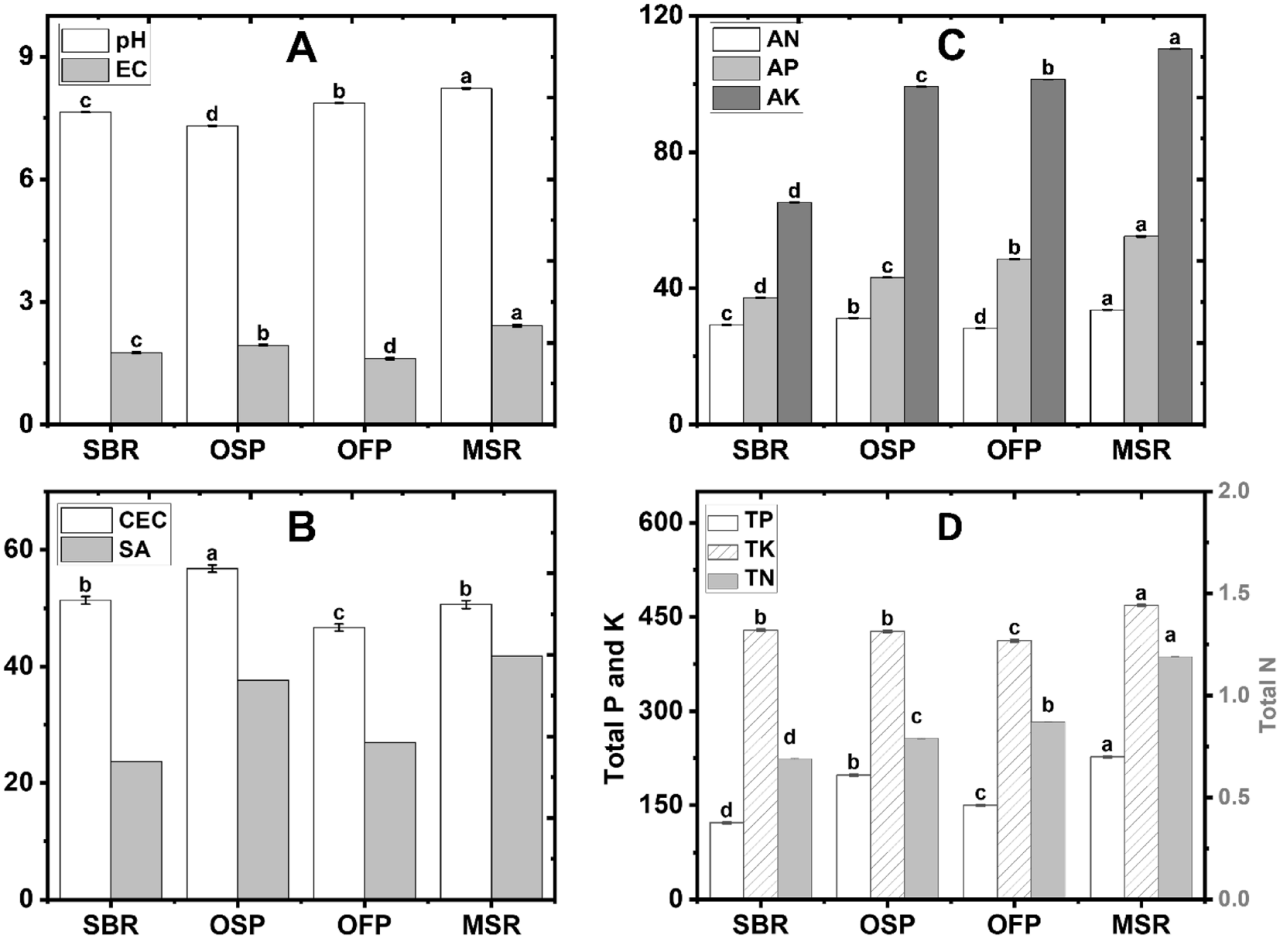
Some characteristics of applied biochars Amendments (Control soil without biochar; SBR sugarcane bagasse residues biochar; OSP olive stone pomace biochar; OFP orange fruit pomace biochar; MSR maize stover residues biochar), including pH and electrical conductivity (EC, dS m^− 1^) (panel **A**), cation exchange capacity (CEC, cmol _(+)_ kg^− 1^) (panel **B**), available N, P and K (mg kg^− 1^) (panel **C**), and total N (g hg^− 1^), K (g kg^− 1^), and P (g kg^− 1^) (panel **D**).

The physicochemical characterization of the biochars evidences a good relationship between surface chemistry, as revealed by FTIR spectra as shown in Fig. 1 and structural development as pointed out by BET surface area (SA) measurements (Fig. 2). Sugarcane bagasse biochar, which has a relatively low surface area of 23.72 m² g⁻¹, still retains abundant oxygenated functionalities such as hydroxyl (O–H) and ether linkages, suggesting incomplete aromatic condensation and prevalence of polar groups that increase the chemical reactivity despite limited porosity. Orange fruit pomace biochar with a slightly higher surface area of 26.97 m² g⁻¹ is enriched in carbonyl (C = O) and carboxylic groups (COOH), which confer strong polarity and adsorption affinity for K even though the modest surface area constrains the overall capacity. In sharp contrast, maize stover biochar shows a substantially greater surface area of 41.82 m² g⁻¹ with FTIR evidence of aromatic C = C domains and residual hydroxyl groups, indicating the occurrence of a more advanced carbonization process which produces a heterogeneous surface combining microporosity with reactive functional sites. The olive stone pomace biochar represents an intermediate case, with 37.64 m² g⁻¹. In general, results emphasize that biochars prepared from different feedstocks have different balances between chemical functionality and physical surface development, and that maize stover residues biochar yield the best compromise between high BET surface area and variety of functional groups for adsorption applications.

Figure 2 illustrates some chemical characteristics of the applied biochar amendments, including SBR, OSP, OFP, and MSR biochars. Properties of the produced biochars varied significantly (*p* < 0.05) based on the type of wastes used, indicating that the raw material plays an important role in biochar quality and functionality. All applied biochars exhibited alkaline pH values (7.31–8.22), reflecting the presence of basic cations and mineral ash accumulated during pyrolysis. MSR biochar resulted the highest pH (8.22), whereas OSP recorded the lowest value (7.31). Similarly, electrical conductivity (EC) values differed significantly, ranging from 1.61 to 2.42 dS m⁻¹, with MSR biochar exhibiting the highest value, suggesting a greater content of soluble salts and mineral nutrients.

Some variations were also recorded in nutrient availability and total macronutrient. More in details, MSR biochar contained significantly higher levels of available N (33.65 mg kg⁻¹), P (55.27 mg kg⁻¹), and K (110.25 mg kg⁻¹), followed by OSP biochar and OFP biochar. Cation exchange capacity (CEC) also ranged from 46.68 to 56.78 cmol_(+)_ kg⁻¹, with OSP biochar showing a significantly higher CEC, implying superior potential for retaining exchangeable cations and enhancing soil nutrient and water-holding capacity. In addition, total nutrient levels (TN, TP, and TK) exhibited similar trends, with MSR biochar again achieving the highest contents, confirming its nutrient enrichment potential.

In general, differences between applied biochars confirm the importance of wastes selection in producing biochar. For example, MSR biochar stood out as the most effective material due to its balanced combination of high nutrient content, compared to o others biochars. In addition, OSP biochar highly excelled in cation exchange capacity. Therefore, these results clearly confirm that the choice of wastes is a critical determinant in producing the most suitable biochar type for specific soil conditions and fertility improvement goals. This also confirms the effective role of biochars derived from MSR and OSP in improving parameters of K showed in the next section.

**Fig. 3:**
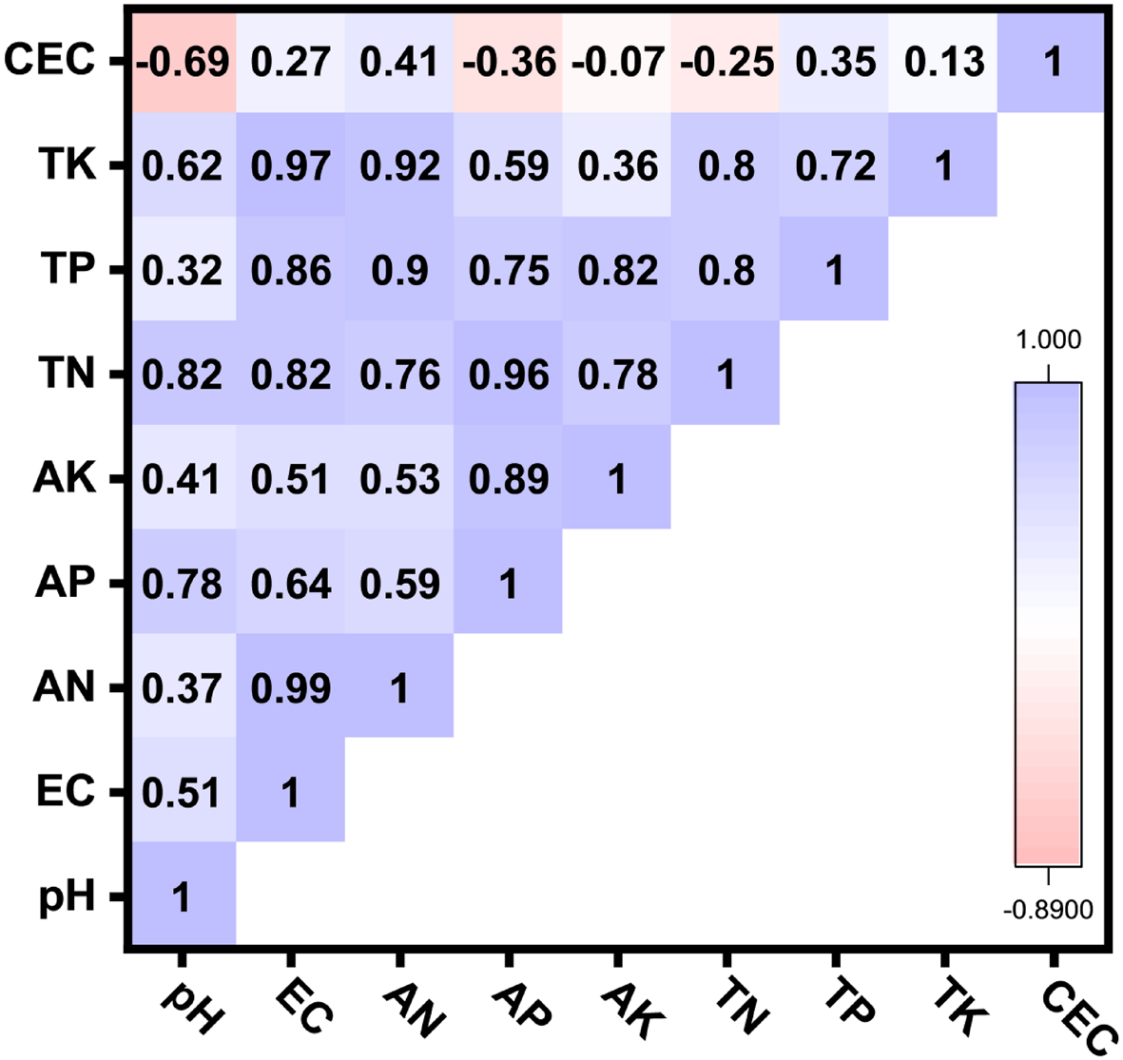
Correlation coefficient matrix of some physicochemical characteristics (pH biochar reaction; EC electrical conductivity; AN available N; AP available P; AK available K; TN total N; TP total P; TK total K; CEC cation exchange capacity) of applied biochars.

Based on the biochar matrix in Fig. 3, EC correlated almost perfectly with available nitrogen (AN, *r* = 0.99) and total potassium (TK, *r* = 0.97). Available phosphorus (AP) correlated strongly with total nitrogen (TN, *r* = 0.96) and available potassium (AK, *r* = 0.89), while the negative correlation between CEC and pH (*r* = − 0.69) indicates that excessive alkalinity may varied exchange capacity through surface charge neutralization. In general, the highly relationships were CEC with K_L_ and PBC^K^ with K_G_, which consistently governed K sorption efficiency and retention across soil types, highlighting the dual importance of exchangeable sites and binding capacity in regulating K availability.

### Influence of Biochar application on some physicochemical characteristics of studied soils

**Fig. 4:**
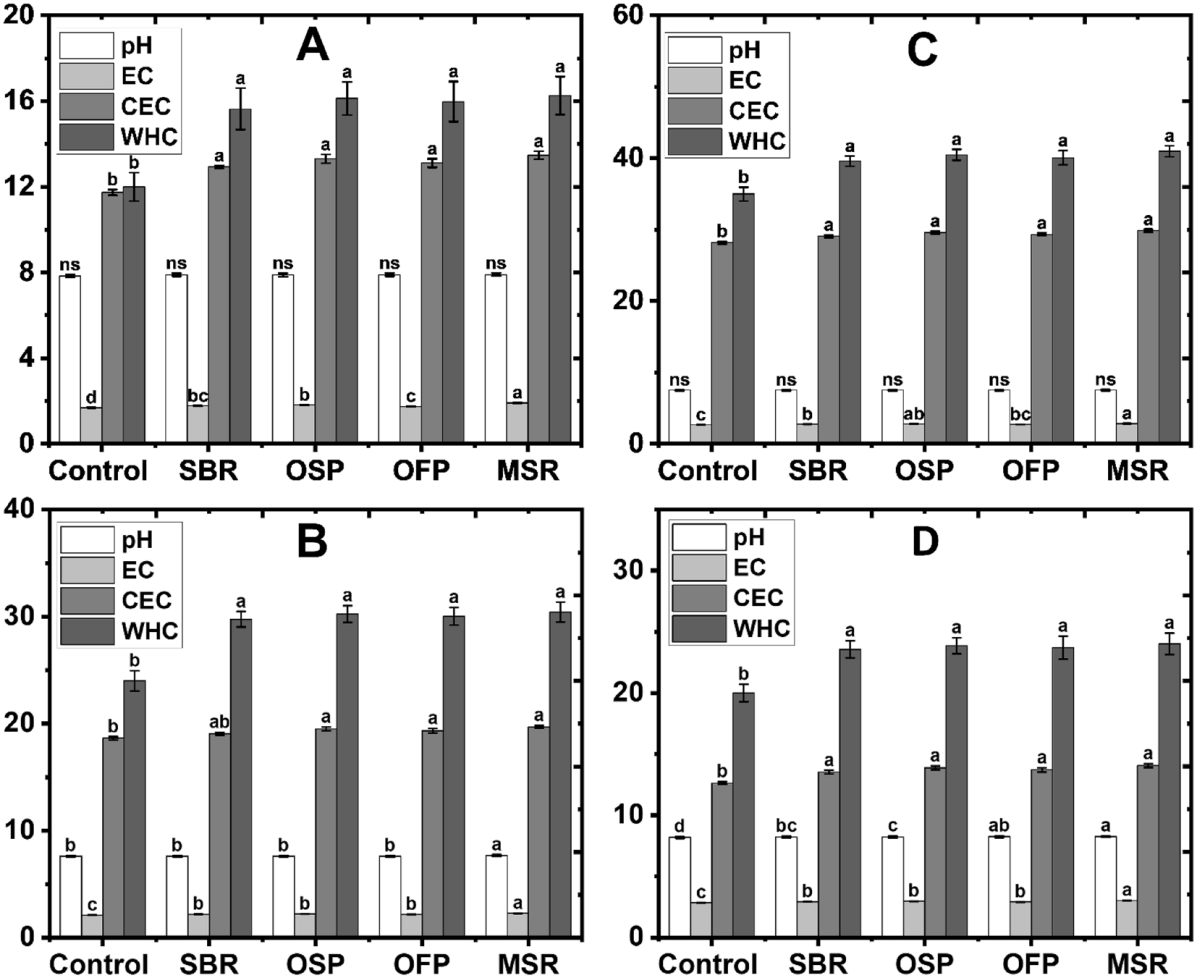
Effects of biochar applications (Control soil without biochar; SBR sugarcane bagasse residue biochar; OSP olive stone pomace biochar; OFP orange fruit pomace biochar; MSR maize stover biochar) at a rate of 3% on some characteristics (pH soil reaction; EC electrical conductivity (dS m^− 1^)); CEC cation exchange capacity (cmol kg^− 1^); WHC water-holding capacity (g hg^− 1^)), of sandy (**A**), loamy (**B**), clayey (**C**) and calcareous (**D**) soils.

The influence of biochars (sugarcane bagasse residue (SBR), olive stone pomace (OSP), orange fruit waste (OFP), and maize stover residues biochar (MSR) biochars) at a rate of 3% on some characteristics of tested soils was evaluated, including soil reaction or acidity (pH), electrical conductivity (EC), cation exchange capacity (CEC), and water-holding capacity (WHC). Figure 4 presents the improvements in some soil characteristics, compared to the unamended treatments. In sandy soil, WHC significantly increased from 12 to 16.26 g hg⁻¹, corresponding to a maximum improvement of 35.5% with MSR biochar. Cation exchange capacity (CEC) also increased from 11.47 to 19.02 cmol_(+)_ kg⁻¹, reflecting a 65.85% increase. Electrical conductivity (EC) increased to 1.91 dS m⁻¹, while pH exhibited a modest increase of 0.89%. Maize stover residues biochar (MSR) was the most effective in the sandy soil, compared to others applied biochars, achieving the highest gains in both WHC and CEC (Fig. 4A). In contrast, loamy soil displayed significant responses to application of biochars. WHC significantly increased by up to 30.43 g hg⁻¹ (26.8%) with MSR biochar, while CEC improved from 18.64 to 24.35 cmol_(+)_ kg⁻¹ (30.7%). Electrical conductivity (EC) value increased to 2.29 dS m⁻¹, and pH increased by 0.79%. MSR biochar Maize stover residues biochar (MSR) performed best in the loamy soil, providing the highest improvement in both WHC and CEC (Fig. 4B). In the clayey soil, WHC improved by 40.98 g hg⁻¹ (17.1%), CEC by 33.25 cmol_(+)_ kg⁻¹ (18.2%), and EC by 2.84 dS m⁻¹ (6.4%), with MSR biochar. Variations of pH values were minimal, not exceeding 0.27%. In addition, MSR biochar outperformed other biochars in clayey soil, achieving the highest WHC and CEC gains (Fig. 4C). Significant responses were observed in the calcareous soil. WHC increased from 20.00 to 24.02 g hg⁻¹ (20.1%), and CEC increased from 12.64 to 33.25 cmol_(+)_ kg⁻¹ (163%) with MSR biochar. Electrical conductivity (EC) increased to 3.03 dS m⁻¹, while pH increased by 0.85%. In general, MSR biochar significantly demonstrated the most effective enhancement in calcareous soil (Fig. 4D).

### Influence of biochars application on the quantity–intensity (Q – I) (ΔK/$\:\mathbf{A}{\mathbf{R}}_{}^{\mathbf{K}}$) relationship and thermodynamic parameters

**Fig. 5:**
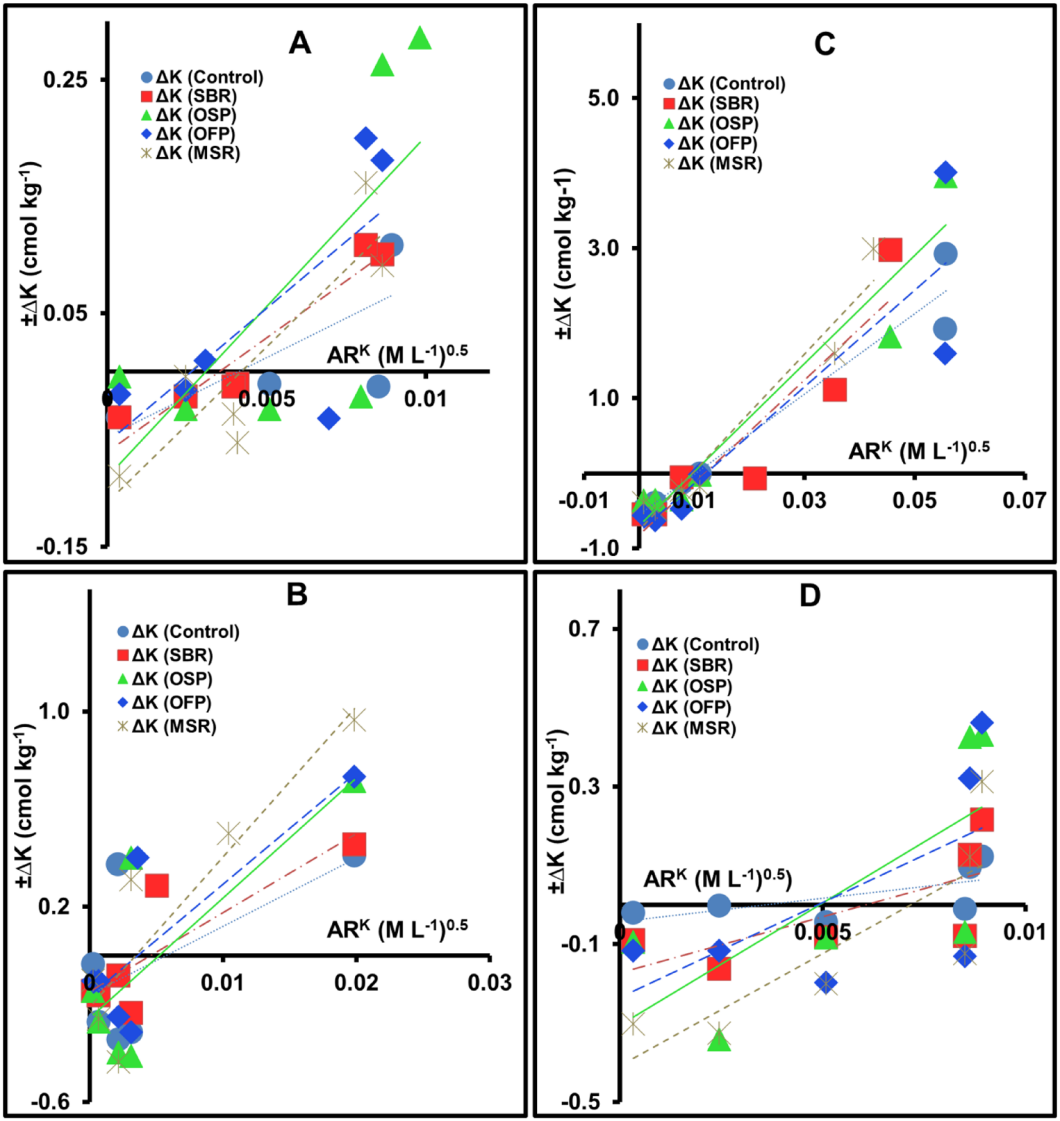
Quantity-intensity (Q–I) relationships of K in soils amended with 3% biochars (Control soil without biochar; SBR sugarcane bagasse residues biochar; OSP olive stone pomace biochar; OFP orange fruit pomace biochar; MSR maize stover biochar; ∆K changing K quantity or quantity factor; AR^K^ activity ratio of K or intensity factor) in sandy (**A**), loamy (**B**), clayey (**C**) and calcareous soils (**D**).

Application of biochar has a clear impact on Q–I (ΔK/$\:{\mathrm{AR}}_{}^{\mathrm{K}}$) relations and K thermodynamic parameters in the investigated soil types, as shown in Fig. 5 and Table 2. Biochars significantly enhanced K thermodynamic parameters (i.e., K_L_, $\:{\mathrm{AR}}_{0}^{\mathrm{K}}$, PBC^K^, ΔG, and K_G_). Most studied soils showed significant improvements in these parameters (Table 2), when compared with unmodified treatments, demonstrating the effect of biochar properties to enhance both the immediate and long-term K availability of plant nutrition.

**Table 2:** Effects of application of biochars (Control soil without biochar; SBR sugarcane Bagasse residues biochar; OSP Olive stone pomace biochar; OFP orange fruit pomace biochar; MSR maize Stover residues biochar) at a rate of 3% on cation exchange capacity (CEC) and thermodynamic parameters of K (CEC cation exchange capacity (cmol_(+)_ kg⁻¹), K_L_ labile-K (cmol kg⁻¹), $\:\mathbf{A}{\mathbf{R}}_{0}^{\mathbf{K}}$ activity ratio of K at equilibrium ((mol L^− 1^)⁻^0.5^), PBC^K^ potential buffering capacity of K (cmol kg⁻¹/(mol L^− 1^)⁻^0.5^)), ΔG Gibbs free energy of K exchange (kcal. mol⁻¹), K_G_ Gabon selectivity coefficient ((L mol⁻¹)) in the sandy, loamy, clayey and calcareous soils (*p value <= 0.05*) .

Treatments	Equation	CEC	−ΔK (K_L_)	$\:{\mathrm{AR}}_{0}^{\mathrm{K}}$	PBC^K^	ΔG	K_G_
*Sandy soil*							
Control	ΔK = 13.544AR^K^ − 0.055	11.470d	0.055c	0.004a	13.544e	− 3.456d	1.181e
SBR	ΔK = 19.601AR^K^ − 0.069	15.036c	0.069c	0.004a	19.601d	− 3.086c	1.304d
OSP	ΔK = 29.249AR^K^ − 0.090	17.023b	0.090b	0.003b	29.249a	− 2.762b	1.718a
OFP	ΔK = 23.010AR^K^ − 0.061	15.010c	0.061c	0.003b	23.010c	− 2.382a	1.533b
MSR	ΔK = 26.681AR^K^ − 0.112	19.023a	0.112a	0.004a	26.681b	− 3.523e	1.403c
*Loamy soil*							
Control	ΔK = 28.036AR^K^ − 0.161	18.640d	0.161c	0.006a	28.036e	− 4.289e	1.504b
SBR	ΔK = 32.327AR^K^ − 0.148	23.645b	0.148c	0.005b	32.327d	− 3.725c	1.367a
OSP	ΔK = 49.168AR^K^ − 0.256	23.425c	0.256a	0.005b	49.168b	− 4.052d	2.099e
OFP	ΔK = 45.757AR^K^ − 0.166	24.254a	0.166c	0.004c	45.757c	− 3.157b	1.887c
MSR	ΔK = 61.647AR^K^ − 0.211	24.352a	0.211b	0.003d	61.647a	− 3.020a	2.531d
*Clayey soil*							
Control	ΔK = 53.584AR^K^ − 0.552	28.123d	0.552e	0.010b	53.584e	− 5.722b	1.905e
SBR	ΔK = 68.777AR^K^ − 0.820	31.256c	0.820a	0.012a	68.777c	− 6.081c	2.200b
OSP	ΔK = 72.047AR^K^ − 0.698	33.621a	0.698d	0.010b	72.047b	− 5.573a	2.143c
OFP	ΔK = 64.405AR^K^ − 0.778	33.256b	0.778b	0.012a	64.405d	− 6.115d	1.937d
MSR	ΔK = 78.174AR^K^ − 0.755	33.252b	0.755c	0.010b	78.174a	− 5.565a	2.351a
*Calcareous soil*							
Control	ΔK = 11.849AR^K^ − 0.043	12.640d	0.043e	0.004d	11.849e	− 3.147a	0.937d
SBR	ΔK = 28.518AR^K^ − 0.172	31.256c	0.172d	0.006b	28.518d	− 4.414d	0.912e
OSP	ΔK = 61.779AR^K^ − 0.304	33.621a	0.304b	0.005c	61.779a	− 3.912c	1.837a
OFP	ΔK = 48.044AR^K^ − 0.234	33.256b	0.234c	0.005c	48.044c	− 3.889b	1.445c
MSR	ΔK = 56.862AR^K^ − 0.408	33.252b	0.408a	0.007a	56.862b	− 4.833e	1.710b

According to obtained results in Table 2, most thermodynamic parameters were significantly affected (*p* < 0.05) by both soil type and biochar waste, showing substantial and statistically meaningful variation among treatments. In sandy soil, K_L_ increased from 0.055 to 0.112 cmol kg⁻¹ (103.6%) with MSR biochar, indicating a significant improvement in available-K. Activity ratio of K ($\:{\mathrm{AR}}_{0}^{\mathrm{K}})$ almost remained stable around 0.003–0.004 (mol L^− 1^)⁻⁰·⁵ with MSR biochar, suggesting unchanged K activity. Potential buffering capacity of K (PBC^K^) increased from 13.544 to 29.25 (cmol kg⁻¹/(mol L^− 1^)⁻⁰·⁵) with OSP biochar (115.96). Gibbs free energy (ΔG) became more negative (from − 3.456 to − 3.523 kcal mol⁻¹) with MSR biochar, while Gabon selectivity coefficient (K_G_) increased from 1.181 to 1.403 L mol^− 1^ (18.8%), reflecting improved K level. Labile-K (K_L_) increased from 0.161 to 0.256 cmol kg⁻¹ (59.01%) with OSP biochar in loamy soil, while $\:{\mathrm{AR}}_{0}^{\mathrm{K}}$ decreased from 0.006 to 0.005 (mol L^− 1^)⁻⁰·⁵, suggesting a more equilibrium with stronger adsorption sites. Potential buffering capacity of K (PBC^K^) increased from 28.036 to 61.647(cmol kg⁻¹/(mol L^− 1^)⁻⁰·⁵) (119.89%) with MSR biochar. ΔG became less negative (from − 4.289 to − 3.020 kcal mol⁻¹), while Gabon selectivity coefficient (K_G_) increased from 1.504 to 2.531 L mol^− 1^ (68.28%), showing higher K retention.

In addition, K_L_ increased from 0.552 to 0.820 cmol kg⁻¹ (48.55%) with SBR biochar in clayey soil, while $\:{\mathrm{AR}}_{0}^{\mathrm{K}}$ slightly increased from 0.010 to 0.012 (mol L^− 1^)⁻⁰·⁵. Potential buffering capacity of K (PBC^K^) also increased from 53.584 to 78.174 (cmol kg⁻¹/ (mol L^− 1^)⁻⁰·⁵) (45.9%) with MSR biochar, compared to the unamended treatment, confirming enhanced PBC^K^ and adsorption strength. Gibbs free energy (ΔG) changed slightly from − 5.722 to − 5.565 kcal mol⁻¹ with MSR biochar, showing nearly unchanged spontaneity but improved energetic balance. Gabon selectivity coefficient (K_G_) increased from 1.905 to 2.351 L mol^− 1^ (23.41%), indicating greater K retention.

In calcareous soil, K_L_ significantly increased from 0.043 to 0.408 cmol kg⁻¹ (849.0%) with MSR, while $\:{\mathrm{AR}}_{0}^{\mathrm{K}}$ rose from 0.004 to 0.007 (mol L^− 1^)⁻⁰·⁵ (75.0%) with MSR biochar, showing a remarkable increase in K exchange and activity. Potential buffering capacity of K (PBC^K^) significantly increased from 11.849 to 61.779 (cmol kg⁻¹ / (mol L^− 1^)⁻⁰·⁵) (421.39%) with OSP biochar. Gibbs free energy (ΔG) became more negative (from − 3.147 to − 4.833 kcal mol⁻¹, by 53.58%) with MSR biochar. Gabon selectivity coefficient (K_G_) increased from 0.937 to 1.710 L mol^− 1^ (82.5%), showing enhanced K retention capacity.

In summary, results showed statistically significant differences among biochar types in all studied soils. The consistent increases in K_L_, $\:{\mathrm{AR}}_{0}^{\mathrm{K}}$, PBC^K^, and K_G_, along with more negative ΔG values, confirm that biochar application enhances both equilibrium and thermodynamic parameters governing K behavior. Maize stover residues biochar (MSR) performed best in most studied soils, OSP biochar was optimal for loamy soil, while OFP biochar was the most effective in the clayey soil. This demonstrates the beneficial effect of MSR and OSP biochars in improving K availability and its thermodynamic parameters in Egyptian soils.

### Influence of applied biochars on wheat biomass and some nutrient uptakes

**Fig. 6:**
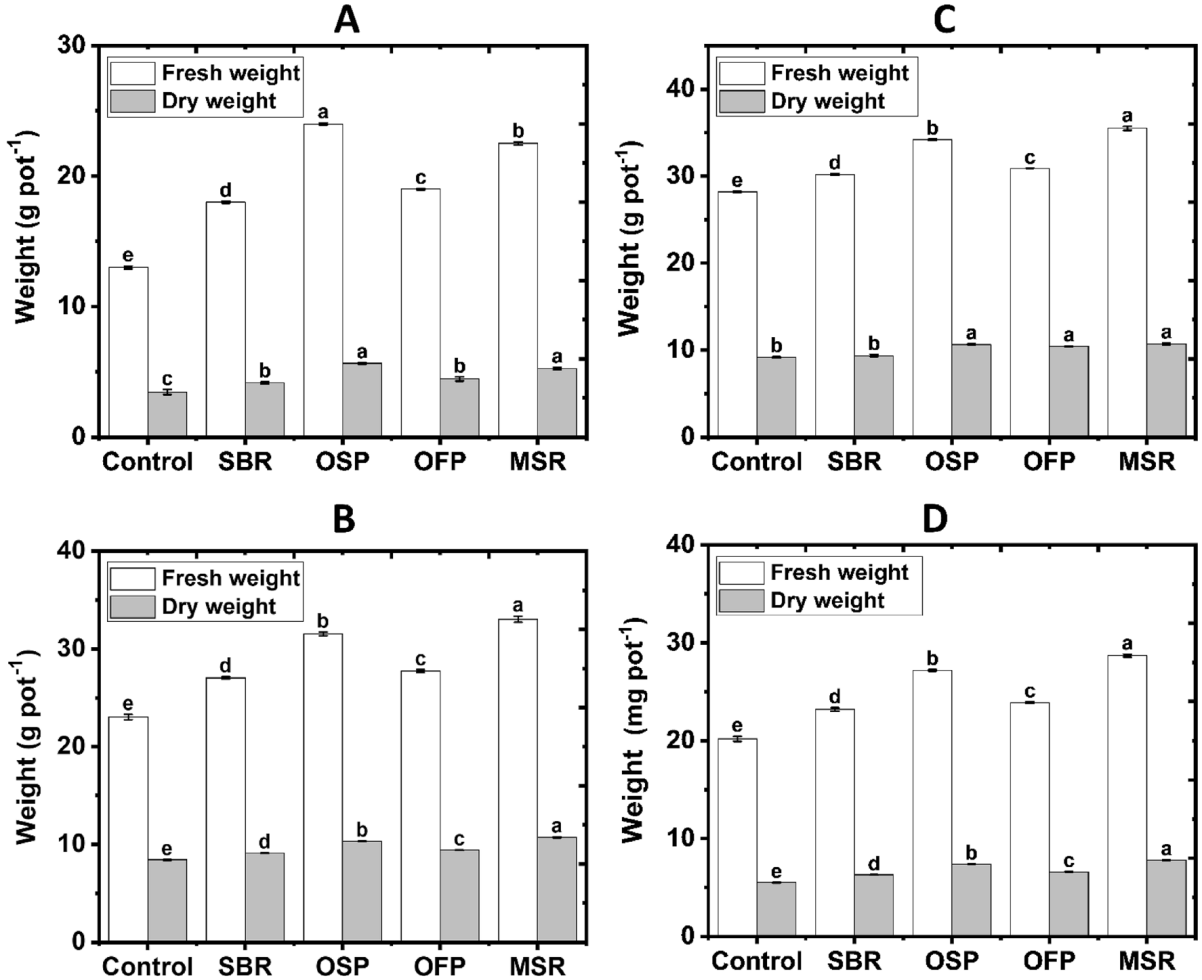
Effects of biochar amendments (Control soil without biochar; SBR sugarcane bagasse residues biochar; OSP olive stone pomace biochar; OFP orange fruit pomace biochar; MSR maize stover residues biochar) at a rate of 3% on the wheat fresh and dry weight in sandy (**A**), loamy (**B**), clayey (**C**) and calcareous soils (**D**).

Biochars significantly enhanced both fresh and dry biomass of wheat plants, compared to the unamended treatment in most studied soils (Fig. 6). The sandy soil with OSP biochar achieved the greatest improvement, with fresh weight increasing by 84.6% and dry weight by 63.8%, compared to the unamended treatment (control), indicating high efficiency under nutrient-poor conditions in this sandy soil. The loamy soil with MSR biochar recorded the highest, with fresh weight increasing by 43.5% and dry weight by 27.3%. In clay soil, MSR biochar was most effective, achieved increase by 25.9% in fresh weight and 16.9% in dry weight. In calcareous soil, MSR also showed superior performance, with fresh weight enhanced by 42.1% and dry weight by 41.8%. In general, soil treated with MSR biochar demonstrated consistent effectiveness in most investigated soils, particularly clay and calcareous soils, while OSP exhibited remarkable superiority in the sandy soil.

**Fig. 7:**
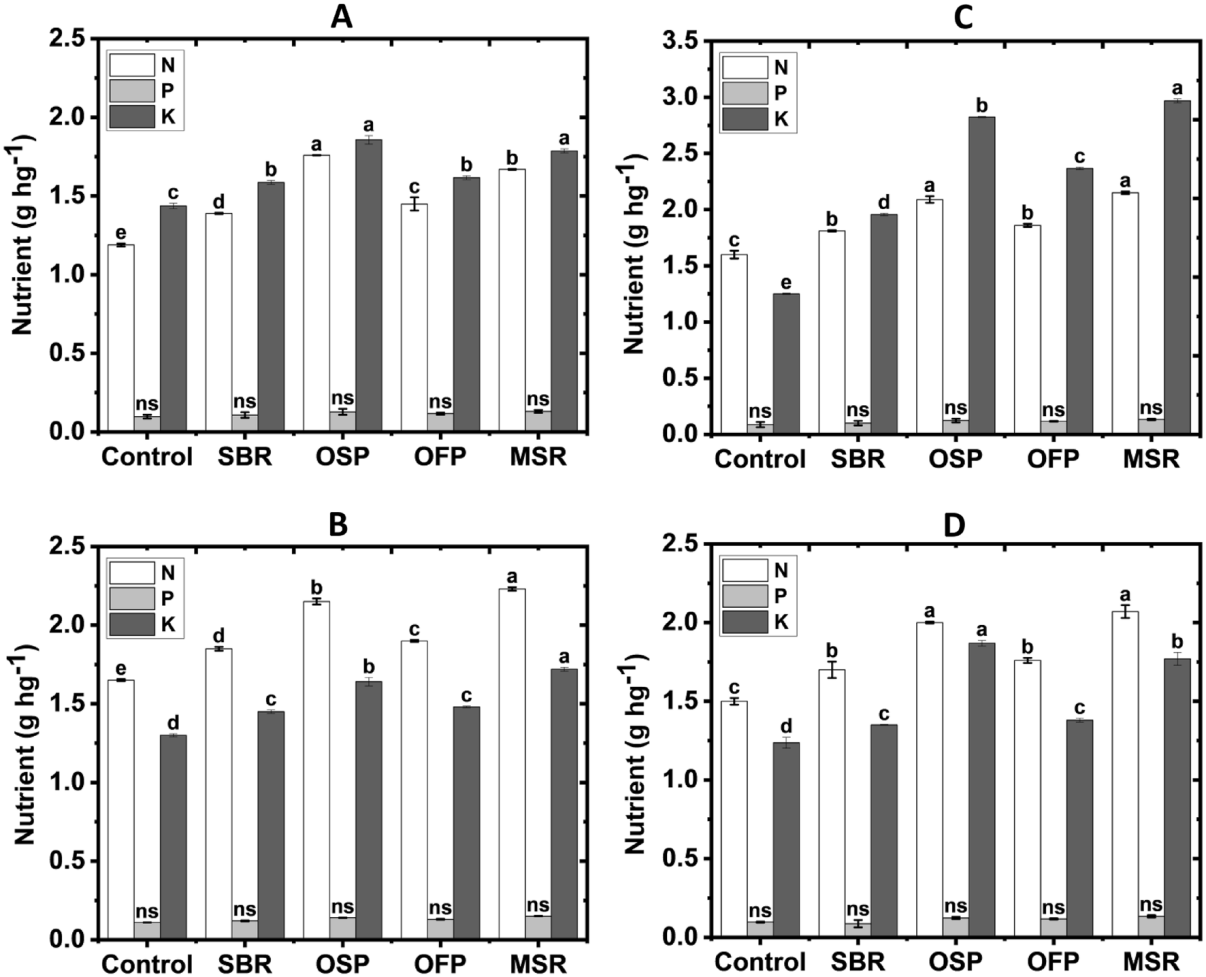
Effects of biochar amendments (Control soil without biochar; SBR sugarcane bagasse residues biochar; OSP olive stone pomace biochar; OFP orange fruit pomace biochar; MSR maize stover residues biochar) at a rate of 3% on nutrient contents (N, P and K) in sandy (**A**), loamy (**B**), clayey (**C**) and calcareous soils (**D**).

Biochar application also led to consistent increases in contents of N, P and K in wheat tissues, compared to the unamended treatment (Fig. 7). In sandy soil, soil with OSP biochar achieved the highest increase, with N rising by 47.9%, P by 30.0%, and K by 29.2%. In loamy sand, the maximum improvement was observed under the MSR biochar, where N increased by 35.2%, P by 36.4%, and K by 32.3%. In clay soil (CS), nutrients were most pronounced under application of MSR, with N rising by 34.4%, P by 27.3%, and K by 137.6%. In calcareous soil, soil with application of OSP showed the greatest improvement in K content (50.8%), while the MSR treatment achieved the highest increases in N (38.0%) and P (40.0%).

**Fig. 8:**
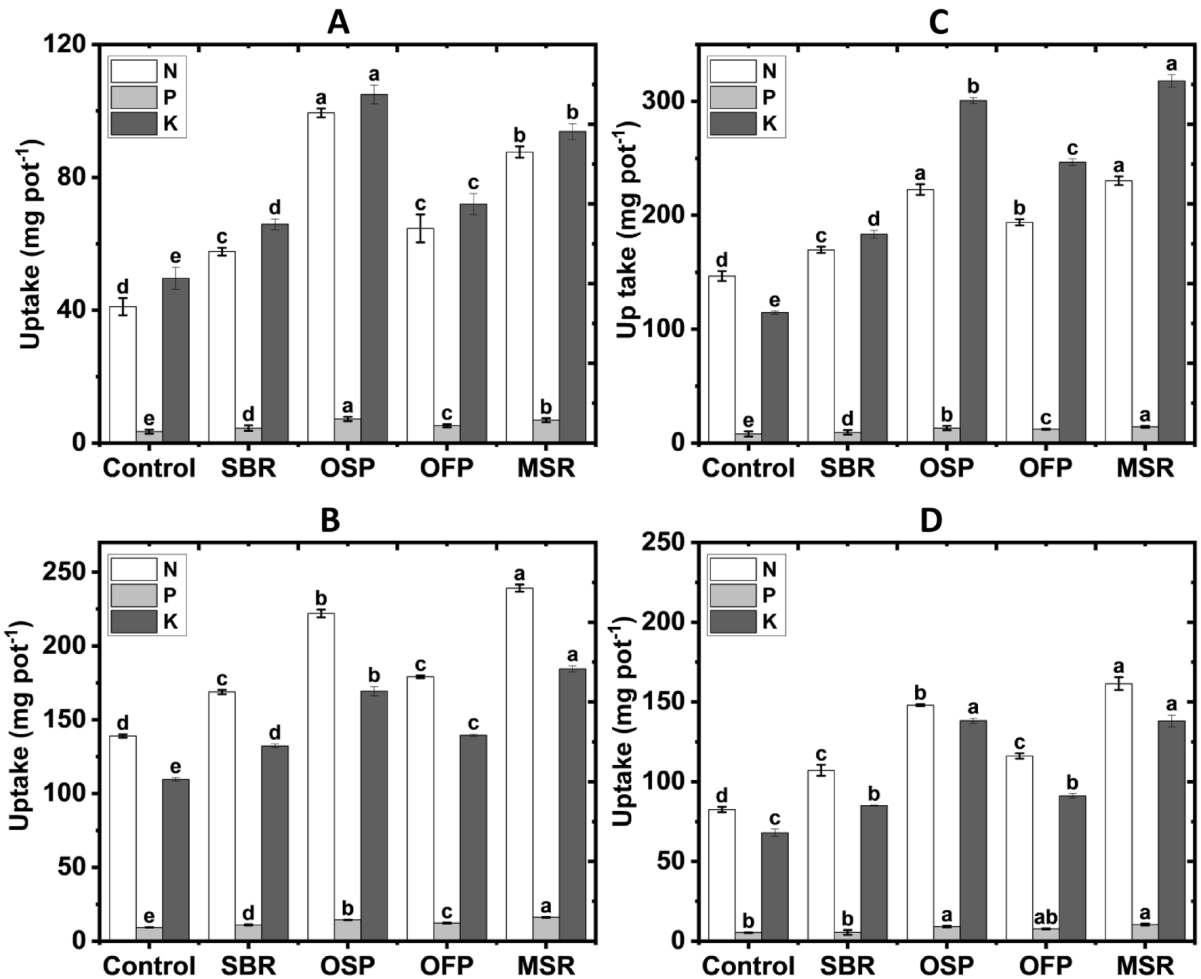
Effects of biochar amendments (Control soil without biochar; SBR sugarcane bagasse residues biochar; OSP olive stone pomace biochar; OFP orange fruit pomace biochar; MSR maize stover residues biochar) at a rate of 3% on some nutrient (N, P and K) uptake by wheat plants grown in sandy (**A**), loamy (**B**), clayey (**C**) and calcareous soils (**D**).

Additionally, uptake of N, P and K by wheat plants further confirmed the beneficial impact of biochar amendments (Fig. 8). In sandy soil, the OSP treatment achieved the highest uptake, with N increasing by 142.2%, P by 113.9%, and K by 111.6%. In loamy sand, the maximum increase was observed under application of MSR biochar, where N rose by 72.1%, P by 73.5%, and K by 68.3%. In clay soil, N, P and K uptake was most pronounced under soil modified with MSR, with N increasing by 57.1%, P by 48.8%, and K by 177.7%. In calcareous soil, soil amended with MSR biochar showed the greatest improvement in N (95.7%) and P (98.5%), while application of OSP achieved the highest K uptake (103.4%). In summary, results highlight that MSR and OSP biochars were the most effective amendments for enhancing N, P and K contents and nutrient uptakes in the studied soils.

### Relations between soil characteristics, thermodynamic parameters of K and wheat growth

**Fig. 9:**
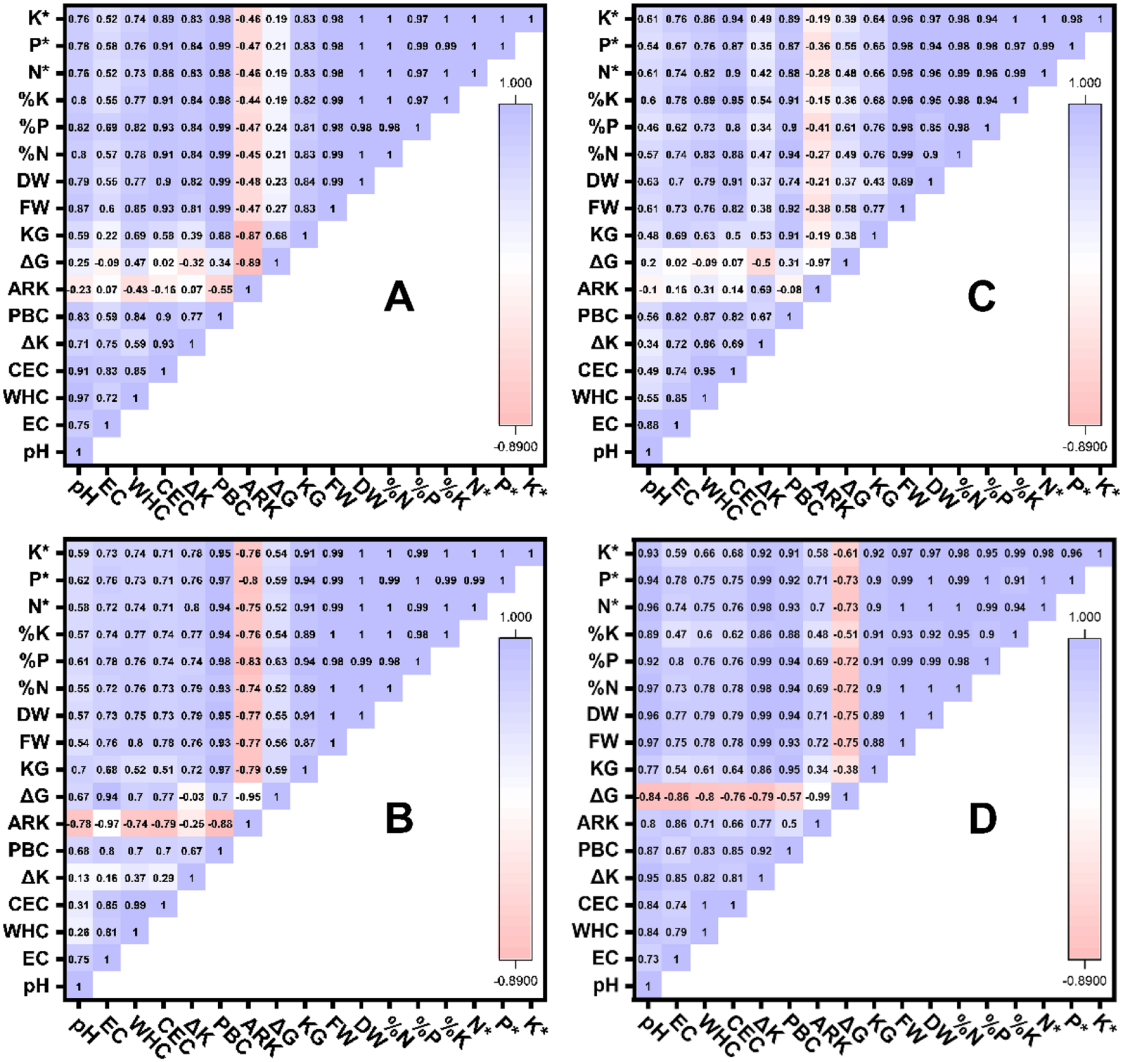
Correlation coefficient matrix of some physicochemical characteristics (pH soil reaction; EC electrical conductivity; WHC water holding capacity; CEC cation exchange capacity) of sandy (**A**), loamy (**B**), clayey (**C**) and calcareous soils (**D**), K dynamic parameters (K_L_ labile-K; $\:{\mathrm{AR}}_{}^{\mathrm{K}}$ activity ratio of K; PBC^K^ potential buffering capacity of K; ΔG Gibbs free energy of K exchange; K_G_ Gabon selectivity coefficient, wheat growth (FW fresh weigh; DW dry weigh), total nutrient contents ( N*, P* and K*) and nutrient uptake ( N, P and K) by wheat plants.

The correlation matrices (Fig. 9) showed several significant relationships between soil characteristics, K thermodynamic parameters and wheat biomass in the sandy soil. Significant positive correlations (*r* ≥ 0.90) were observed between CEC and both ΔK (*r* = 0.93) and PBC^K^ (*r* = 0.90), indicating that the enhancement of soil exchange capacity directly increased K release. PBC^K^ showed nearly correlations with plant growth parameters, including fresh weight (*r* = 0.99), dry weight (*r* = 0.99), and wheat nutrient contents (N, P, and K; all *r* ≈ 0.99), highlighting its dominant role in sustaining K availability and promoting plant nutrition. Similarly, ΔK was closely related to CEC (*r* = 0.93) and WHC (*r* = 0.59). In contrast, $\:{\mathrm{AR}}_{0}^{\mathrm{K}}$ exhibited strong negative correlations with PBC^K^ (*r* = − 0.55) and K_G_ (*r* = − 0.87), while ΔG was negatively correlated with $\:{\mathrm{AR}}_{0}^{\mathrm{K}}$ (*r* = − 0.89) and positively associated with K_G_ (*r* = 0.68), suggesting that K sorption processes were thermodynamically favorable.

In the loamy soil, correlation analysis showed significant relationships among soil characteristics, K-dynamics parameters, and wheat biomass. The potential buffering capacity of K (PBC^K^) exhibited exceptionally high positive correlations with K_G_ (*r* = 0.97), P in wheat (*r* = 0.98), and plant growth indicators such as fresh weight (*r* = 0.93) and dry weight (*r* = 0.95), emphasizing its crucial role in regulating K availability and sustaining plant productivity. Similarly, PBC^K^ was strongly associated with CEC (*r* = 0.70) and EC (*r* = 0.80), suggesting that enhanced soil charge and ionic strength contributed to higher K retention capacity. The equilibrium activity ratio of K ($\:{\mathrm{AR}}_{0}^{\mathrm{K}}$) showed pronounced negative correlations with EC (*r* = − 0.97), PBC^K^ (*r* = − 0.88), and ΔG (*r* = − 0.95), reflecting the thermodynamically favorable nature of K adsorption and stabilization in the soil matrix. Moreover, ΔG exhibited a strong positive association with EC (*r* = 0.94) and CEC (*r* = 0.77), confirming that improvements in soil ionic environment enhanced K sorption energy. Wheat growth and nutrient parameters were also highly interrelated (*r* ≥ 0.98) and tightly linked to PBC^K^ and K_G_ (*r* ≥ 0.94), demonstrating that potential buffering capacity of K and kinetic behavior directly influenced nutrient uptake and biomass production.

In the clayey soil, CEC showed strong correlations with WHC (*r* = 0.95), K in wheat (*r* = 0.95), and dry weight (*r* = 0.91). The potential buffering capacity of K (PBC^K^) exhibited strong positive relationship with K_G_ (*r* = 0.91), fresh weight (*r* = 0.92), N in wheat (*r* = 0.94), and K in wheat (*r* = 0.91), indicating that enhanced PBC^K^ was directly linked to improved plant nutrition and growth under clayey conditions. ΔK was moderately correlated with WHC (*r* = 0.86) and EC (*r* = 0.72). Negative correlations were observed between $\:{\mathrm{AR}}_{0}^{\mathrm{K}}$ and most growth-related parameters, though weaker than in other soil types, while ΔG was inversely related to ΔK (*r* = − 0.50) and $\:{\mathrm{AR}}_{0}^{\mathrm{K}}$ (*r* = − 0.97). Plant nutrient content and uptakes were almost perfectly correlated (*r* ≥ 0.98) and strongly associated with PBC^K^, CEC, and K content in wheat (*r* ≥ 0.94).

In the calcareous soil, ΔK correlated strong relations with pH (*r* = 0.95), EC (*r* = 0.85), and PBC^K^ (*r* = 0.92). PBC^K^ was also highly correlated with CEC (*r* = 0.85), K_G_ (*r* = 0.95), and ΔK (*r* = 0.92), emphasizing its key role in maintaining K equilibrium under calcareous conditions. Plant growth indicators, including fresh and dry weights, were nearly perfectly associated with ΔK (*r* = 0.99), PBC^K^ (*r* = 0.93–0.94), and pH (*r* ≈ 0.97). Similarly, nutrient concentrations and uptake (N, P, and K) exhibited extremely high correlations (*r* ≥ 0.97) and strong positive relationships with PBC^K^ and ΔK (*r* ≥ 0.93). In contrast, ΔG showed strong negative correlations with $\:{\mathrm{AR}}_{0}^{\mathrm{K}}$ (*r* = − 0.99), ΔK (*r* = − 0.79), and PBC^K^ (*r* = − 0.57).

## Discussion

### Characteristics of studied soils

The improvements in soil characteristics due to biochar application at a rate of 3% are consistent with most previous studies highlighting biochar’s multifunctional role in enhancing soil fertility. Biochar also improves soil structure, increases CEC, enhances nutrient availability (N, P, K) and WHC, especially in coarse and medium-textured soils^1,16,24,34^. Its highly porous structure and abundance of oxygen-containing functional groups facilitate nutrient adsorption and reduce leaching losses, thereby improving nutrient use efficiency^19,35^.

In addition, biochar contributes to greater soil aggregation and microbial activity, which in turn promote nutrient cycling and soil resilience under stress conditions^5,36^. These physicochemical and biological enhancements together underpin the positive effects observed in this study on K availability and retention in tested soils.

Moreover, biochar application significantly increased most physical and chemical characteristics of soils. For example, in this study, CEC increased by 65.9% in sandy soil with MSR biochar, while in loamy soil, CEC increased by 30.6% with MSR biochar. In clayey soil, CEC increased by 19.5% with OSP biochar, while in calcareous soil, the increase was observed, from 12.640 to 33.621 cmol_(+)_ kg⁻¹ (165.9%) with OSP biochar. These increases in the sandy and calcareous soils may be due to the poor content of the soils of adsorption complex like organic matter and clay contents, while in the clay soil (Vertical) which contain about 50% clay (Table 1). This also indicates that biochar is particularly effective in soils with low initial fertility and high calcium carbonate content, where K retention is limited^35^. These results corroborate previous results that biochar increases the number of active exchange sites, especially in coarse-textured and soil poor in adsorption complexes.

### Thermodynamic parameters of K in the studied soils

The thermodynamic parameters of K in tested soils were improved due to biochar application, and can be attributed to both the physical and chemical characteristics of applied biochars and their interactions with soil components. Each parameter, including CEC, K_L_, $\:{\mathrm{AR}}_{0}^{\mathrm{K}}$, PBC^K^, ΔG, and K_G_, provides complementary information about the availability, immediate activity, retention, and thermodynamic favorability of K in soils.

The increases of K dynamics to biochar application varied considerably between the studied soils, reflecting the interaction between soil characteristics and biochar characteristics.

These improvements can be attributed to the ability of biochar to compensate for low fertility, increase surface area, and provide additional exchange sites. Soil-specific trends indicate that coarse-textured or Ca-rich soils respond more to alkaline, nutrient-rich biochars, emphasizing the importance of matching biochar type to soil characteristics^5,34,35,37^. Furthermore, the high porosity and surface area of biochars improved physical retention and provided diffusion pathways for K ions, increasing their residence time in the soil solution^1,35^. The abundance of oxygen-containing functional groups, such as carboxyl, hydroxyl, and phenolic moieties, created additional cation exchange sites for K⁺ adsorption and desorption^1^. The content of K in wastes also plays an effective role in determining K in biochar, as reflected in the higher $\:{\mathrm{AR}}_{0}^{\mathrm{K}}$ values observed for biochars such as MSR and OSP biochars. In calcareous soils, the exchange sites of biochars helped mitigate K fixation by competing with Ca²⁺ ions thereby enhancing the mobility and availability of K. Moreover, biochar–soil interactions strongly influenced both kinetic and K thermodynamic behavior. Gibbs free energy (ΔG) generally indicated thermodynamically favorable K sorption and release processes^5,20,36,38^.

#### The labile-K (K_L_)

Labile-K (K_L_) values improved as a result of the application of biochars, reflecting the rate at which exchangeable K becomes available for plant uptake^1,2^. The observed increase in K_L_ = 0.112 cmol kg⁻¹ in sandy soils with the MSR biochar, 0.256 cmol kg⁻¹ in loamy soils with OSP biochar, 0.755 cmol kg⁻¹ in clayey soils with MSR biochar, and 0.408 cmol kg⁻¹ in calcareous soils with MSR biochar. The remarkable increases in K_L_ in calcareous soils can be explained by the enhanced adsorption sites, greater surface area, and abundant functional groups provided by MSR or OSP, which are rich in K. Soils of higher CEC thus showed higher K_L_ values, which reflected increased diffusion and desorption of biochar. In contrast, low CEC soils had relatively lower K_L_, reflecting strong adsorption of K⁺ onto mineral and organic colloids^39^.

Variations in K_L_ between soils can also be explained by variations in physical, chemical and mineralogical properties of studied soils, such as soil texture, mineralogical composition, organic matter, CEC, available nutrients and presence of competing cations such as Ca²⁺ and Mg²⁺ influencing the ion exchange equilibrium^40,41^. Values of Labile-K (K_L_) as observed correspond to the readily exchangeable fraction of soil K that can be mobilized under equilibrium conditions, as indicated by the Q – I relationship curve^39^. Application of biochar increased the number of active exchange sites in sandy soils, thereby increasing the rate of release of K⁺. In clayey soils, even though K_L_ was already high, the addition of biochar further facilitated K desorption by adding more exchange sites and thus increasing the surface area, in line with observations made on loamy and calcareous soils^3,21,36^.

In general, these findings tend to elucidate that K_L_ is not just a measure of K availability but also reflects the interplay between soil physicochemical characteristics and biochar characteristics. The enhancements in K_L_ following biochar application prove that biochars can enhance both the immediate and potential availability of K, mainly in soils of low initial fertility or with limited mobility of K⁺^3,40,42,43^.

#### Activity ratio of K at equilibrium ($\:\mathbf{A}{\mathbf{R}}_{0}^{\mathbf{K}}$)

The activity ratio of K at equilibrium $\:\left({\mathrm{AR}}_{0}^{\mathrm{K}}\right)$, derived from the Q – I relationship (line intersects the activity ratio (X or I axis) axis), represents the immediate pool of exchangeable K in the soil that is readily available for plant uptake (according to the Davis’s equation Eq. 2 and calculated activity of K by Eq. 3). Soil samples were equilibrated with CaCl₂ solutions containing different amounts of KCl, and $\:{\mathrm{AR}}_{}^{\mathrm{K}}$ was calculated by comparing the K concentration in the soil solution to that in the soil. The $\:{\mathrm{AR}}_{0}^{\mathrm{K}}$ values increased in all soils following biochar application at a rate of 3%, indicating enhanced K availability. Specifically, $\:{\mathrm{AR}}_{0}^{\mathrm{K}}$ almost remained stable by 0.004 (mol L^− 1^)⁻⁰·⁵ in sandy soil with the MSR biochar, while in loamy soil decreased to 0.003 (mol L^− 1^)⁻⁰·⁵ with the MSR biochar, a significant increase from 0.010 to 0.012 mol L⁻¹ in clayey soil with the SBR and OFP biochars, and 0.007 (mol L^− 1^)⁻⁰·⁵ in calcareous soil with the MSR biochar. Calcareous soil exhibited the highest increase (75%) with the MSR biochar, whereas clayey soil showed minimal change, likely due to its higher exchangeable-K sites and stronger adsorption capacity. Differences of $\:{\mathrm{AR}}_{0}^{\mathrm{K}}$ in the studied soils may also be influenced by variations in CEC value, EC value, value of ionic strength (I, Eq. 1) and the concentrations of some nutrients i.e. Ca^2+^ and Mg^2+^ ions in the soil solutions, which affect the immediate activity of K^3,40,43^. These results suggest that biochar application can enhance the labile-K pool, readily available-K, and generally improve K availability, especially in soils with lower initial fertility or coarse textures.

#### Potential buffering capacity of K (PBC^K^)

Potential buffering capacity of K (PBC^K^) values of the studied soils, representing the slope of the linear portion of the Q/I or (ΔK/ $\:{\mathrm{AR}}_{0}^{\mathrm{K}}$ ) plot (Eq. 5), increased following biochar addition. In sandy soil, PBC^K^ increased to 29.249 (cmol kg⁻¹/(mol L^− 1^)⁻⁰·⁵) with the OSP biochar, in loamy soil to 61.647 (cmol kg⁻¹/(mol L^− 1^)⁻⁰·⁵) with the MSR biochar, in clayey soil to 78.174 (cmol kg⁻¹/(mol L^− 1^)⁻⁰·⁵) with the MSR biochar, and in calcareous soil to 61.779 (cmol kg⁻¹/(mol L^− 1^)⁻⁰·⁵) with the OSP biochar. The great increases in PBC^K^ in calcareous soils can be attributed by the improved adsorption sites, high surface area, and abundant functional groups provided by MSR or OSP. Potential buffering capacity of K (PBC^K^) values can be classified as very low (< 20), low (20–50), medium (50–100), elevated (100–200), and high (> 200) (cmol_(+)_ kg⁻¹/(mol L⁻⁰·⁵)), according to classification by Zharikova^44^. Most of the soils studied belongs to the low to medium range, and this can be attributed to variations in K sites, soil texture, organic matter content, and mineral composition.

A relatively high PBC^K^ values indicate a greater soil capacity to buffer against K⁺ depletion due to plant uptake or leaching, maintaining a relatively stable K concentration in the soil solution over time^3^. Conversely, low PBC^K^ soils have limited buffering capacity, which results in faster depletion of K⁺ under crop removal or irrigation^2^. The observed increases in PBC^K^ following biochar application suggest that biochars enhance the PBC^K^ or ability to adsorb and retain K ions, likely due to their high surface area, porosity, and functional groups. When comparisons of these increases after the application of the biochars applied in the studied soil, we note that these thermodynamic parameters of K have significantly improved, compared to the unmodified treatments. This is consistent with the improvements caused by the application of biochar in most previous studies referenced in our study.

Additionally, differences in PBC^K^ of studied soils can also be linked to inherent soil characteristics. For example, with low CEC and coarse texture in sandy soils, typically exhibit lower PBC^K^, while clayey soils, which have higher CEC and more exchangeable sites, show higher PBC^K^. Calcareous soils may experience moderate increases due to the interplay between biochar and CaCO_3_ content, which influences K⁺ fixation and exchange dynamics. Additionally, variations in organic matter content among soils can modify PBC^K^ by affecting both exchange sites and the complexation of K ions^3,40,41^.

In summary, these results highlight the role of biochar as an amendment that improves soil nutrients levels, especially K, thereby enhancing nutrient retention and mitigating rapid fluctuations in K availability. These improvements are particularly important for coarse-textured or low-fertility soils, where natural K retention is limited. This suggests that the successive application of biochar can reduce the risk of K deficiency, improve fertilizer use efficiency, and support sustainable crop production in diverse soil environments.

### Gibbs free energy of K exchange (ΔG)

Gibbs Free Energy (ΔG) became more negative following biochar application with the MSR biochar (Eq. 6), up to − 3.523 kcal mol⁻¹ in sandy, − 3.020 kcal mol⁻¹ in loamy, − 5.565 kcal mol⁻¹ in clayey, and − 4.833 kcal mol⁻¹ in calcareous soils, indicating more thermodynamically favorable K exchange. The Gibbs free energy of K exchange (ΔG) serves as an index of the chemical potential driving the movement of K ions from soil solids to the soil solution and ultimately to plant roots. More negative ΔG values indicate that the release of K⁺ from soil exchange sites is thermodynamically favorable, facilitating higher availability for plant uptake.

In this study, the observed variation in ΔG in studied soils can be attributed to differences in CEC, clay mineralogy, and the abundance of exchangeable K⁺. Soils with higher CEC and more loosely bound K, such as clayey soils, exhibited the most negative ΔG (–5.565 kcal mol⁻¹), reflecting a greater tendency for spontaneous K release. Conversely, sandy soils with lower CEC showed less negative ΔG (–3.523 kcal mol⁻¹), indicating more limited K mobility. These findings align with previous reports that soils rich in exchangeable K tend to show lower Gibbs free energy values^3,45,46^, and that negative ΔG values correlate with spontaneous K exchange, whereas less negative ΔG suggests stronger K binding and reduced release^47^.

The changes in ΔG also reflect the influence of biochar type and application, as biochar increases available exchange sites, enhances soil surface area, and interacts with soil minerals to facilitate K desorption. Therefore, the application of nutrient-rich biochars not only increases K availability but also makes the exchange process more thermodynamically favorable, ensuring sustained K supply over time^43^.

#### Gabon selectivity coefficient (K_G_)

The Gabon selectivity coefficient (K_G_) provides a quantitative measure of the soil’s selectivity for K^+^ ions (Eq. 7) over competing cations such as Ca²⁺ and Mg²⁺ under equilibrium conditions, reflecting both the chemical affinity and the availability of exchange sites in the soil. Higher K_G_ values indicate a stronger tendency of the soil to retain K⁺, which is closely related to the mineralogical composition, cation exchange capacity (CEC), and the presence of planar adsorption sites on clay minerals^3,21,48,49^.

In this study, K_G_ values varied among soils and biochar treatments, ranging from 0.937 in untreated calcareous soil to 2.531 in loamy soil treated with the MSR biochar. Sandy soils generally exhibited moderate K_G_ values (1.181–1.403), whereas clayey and loamy soils amended with nutrient-rich biochars showed higher K_G_, consistent with their greater CEC and enhanced K⁺ retention capacity. These variations demonstrate that biochar addition modifies the selectivity behavior of soils, increasing their ability to retain K while reducing potential losses through leaching or fixation.

The fluctuations in K_G_ values can be largely attributed to the amounts of exchangeable Ca²⁺ and Mg²⁺, as these cations compete with K⁺ for exchange sites^41^. Additionally, the preferential attraction of K⁺ ions to planar positions on clay colloids contributes to higher selectivity coefficients, particularly in soils with high clay content or well-developed mineral surfaces^43^. By enhancing K_G_, biochar application not only improves K retention but also facilitates a more stable and sustainable K supply for crops, which is especially beneficial in coarse-textured or low-fertility soils where natural K buffering is limited. These results align with reported ranges of K_G_ in other studies, which varied from 2.30 to 51.9 depending on soil type, exchangeable cation concentrations, and mineralogy^39,48,49^.

In general, the findings confirm that biochar amendments significantly enhance K dynamics in soils through complementary mechanisms. The increase in CEC expanded the number of available exchange sites, while higher K_L_ values indicated accelerated K release from biochar surfaces. The elevated $\:{\mathrm{AR}}_{0}^{\mathrm{K}}$ and PBC^K^ values demonstrate improved immediate and sustained K availability, respectively, ensuring both short- and long-term nutrient supply. Furthermore, the negative ΔG values confirm that K exchange processes became more thermodynamically favorable following biochar application. These integrated effects highlight the critical role of feedstock selection and soil matching in optimizing biochar performance. The results emphasize that biochar not only acts as a nutrient source but also as a functional amendment that regulates ion exchange, energy balance, and nutrient buffering in diverse soil environments.

### Wheat growth and nutrient uptakes

The substantial enhancements in soil physicochemical characteristics and K dynamics following biochar application have direct and meaningful implications for wheat growth and nutrient acquisition across different soil types. In sandy soils, the marked increase in WHC and CEC under MSR and OSP treatments improved soil moisture retention and exchangeable cation availability, which are critical for sustaining wheat growth under nutrient-poor conditions. The simultaneous enhancement of K_L_, PBC^K^, and K_G_, along with more favorable ΔG values, facilitated greater K mobility and retention, thereby supporting higher nutrient uptake and biomass accumulation. These findings are consistent with the observed increases in wheat fresh and dry weights, as well as N, P, and K concentrations in plant tissues, highlighting the role of biochar in mitigating the limitations of coarse-textured soils^19,21,35^.

In loamy and clayey soils, moderate improvements in CEC, WHC, and K PBC^K^ enhanced the availability of both macro- and micronutrients, promoting steady nutrient supply and sustaining plant metabolic activity. Specifically, the strong positive correlations between PBC^K^, K_G_, and wheat nutrient content indicate that biochar-mediated enhancement of PBC^K^ directly translates into improved nutrient assimilation efficiency. This aligns with previous reports suggesting that biochar additions can stabilize K release, enhance nutrient retention, and support plant growth even in soils with higher clay content or moderate fertility levels^3,36^.

For calcareous soils, biochar applications effectively mitigated the negative effects of high pH on nutrient availability. The alkalinity and mineral content of biochars, particularly MSR and OSP, likely reduced K fixation by competing with Ca²⁺ for exchange sites, thereby increasing labile-K pools and improving the thermodynamic favorability of K release (ΔG became more negative). This improved K availability was closely associated with enhanced wheat biomass and higher N, P, and K uptake, demonstrating that biochar can overcome the limitations imposed by calcium carbonate-rich soils^34,43^.

In general, recorded enhancements indicate that the choice of biochar type is crucial for maximizing wheat growth and nutrient uptake. MSR biochar has a high nutrient content, consistently promoted N and P accumulation, while OSP biochar was particularly effective in enhancing K availability in sandy and calcareous soils. These soil-specific responses emphasize the importance of tailoring biochar amendments to soil characteristics to optimize nutrient use efficiency, improve crop productivity, and reduce dependence on chemical fertilizers, thereby contributing to sustainable agricultural management^1,5,50^.

In final, these studied thermodynamic parameters provide clear valuable insights and long-term predictions of K dynamics in biochar-improved studied soils. They also reveal the effective role of biochar in enhancing most physical and chemical characteristics of soils, especially in increasing the number of active exchange sites, thereby improving the soil’s ability to supply K sustainably. The consistency of these observations across sandy, loamy, clayey, and calcareous soils confirms that biochar applications can be tailored to soil type and initial fertility status, maximizing K use efficiency and potentially reducing the need for frequent K fertilization. This reduces fertilizer costs and consumption, and promotes environmental sustainability. It also demonstrates the importance of choosing sustainable strategic tools, such as biochar, to enhance K levels in common Egyptian soils in an environmentally friendly manner, while reducing the accumulation or burning of these agricultural wastes.

## Conclusions

The application of biochar at a rate of 3% (w/w) derived from various agricultural wastes significantly improved K dynamics and other properties in the studied Egyptian soils of varying textures. Maize stover residues biochar (MSR) and olive stone pomace (OSP) biochars were the most effective, increasing CEC, WHC, K_L_, $\:{\mathrm{AR}}_{0}^{\mathrm{K}}$, PBC^K^, and K_G_ while decreasing ΔG, thus confirming spontaneous and energy-efficient K exchange interactions. The response varied with soil texture, with MSR generally performing best in all studied soils, followed by OSP biochar. These results demonstrate that matching the type of biochar to the soil properties can enhance K thermodynamic, availability, nutrient holding, and crop productivity at long-term. Therefore, biochar represents a practical, sustainable, and eco-environmentally approach to improving soil fertility, reducing unsafe disposal or accumulation of waste, chemical fertilizer use, and recycling agricultural waste to promote nutrient use efficiency in arid and semi-arid regions.

## Data Availability

All data of this study are available from the corresponding author upon reasonable request.
